# The Evolutionary Scenario of Pediatric Unclassified Primary Antibody Deficiency to Adulthood

**DOI:** 10.3390/jcm12134206

**Published:** 2023-06-22

**Authors:** Mayla Sgrulletti, Giorgio Costagliola, Giuliana Giardino, Simona Graziani, Elisabetta Del Duca, Silvia Di Cesare, Gigliola Di Matteo, Rita Consolini, Claudio Pignata, Viviana Moschese

**Affiliations:** 1 Pediatric Immunopathology and Allergology Unit, Policlinico Tor Vergata, University of Tor Vergata, 00133 Rome, Italy; maylasg@gmail.com (M.S.); simonagraziani@hotmail.com (S.G.); elisabetta.delduca@ptvonline.it (E.D.D.); 2 Ph.D. Program in Immunology, Molecular Medicine and Applied Biotechnology, University of Rome Tor Vergata, 00133 Rome, Italy; 3 Section of Clinical and Laboratory Immunology, Division of Pediatrics, Department of Clinical and Experimental Medicine, University of Pisa, 56126 Pisa, Italy; giorgio.costagliola@hotmail.com (G.C.); rita.consolini@med.unipi.it (R.C.); 4 Pediatric Section, Department of Translational Medical Sciences, Federico II University, 80131 Naples, Italy; giuliana.giardino@unina.it (G.G.); pignata@unina.it (C.P.); 5 Department of Systems Medicine, University of Tor Vergata, 00133 Rome, Italy; di.cesare@med.uniroma2.it (S.D.C.); di.matteo@med.uniroma2.it (G.D.M.)

**Keywords:** unclassified primary antibody deficiency, primary antibody deficiency, transient hypogammaglobulinemia of infancy, children, inborn errors of immunity, TNFRSF13B mutations, common variable immunodeficiency, selective IgA deficiency, isolated IgM deficiency

## Abstract

Background: Unclassified primary antibody deficiency (unPAD) is a relatively novel inborn error of immunity (IEI) condition that can vary with time to more defined entities. Since long-term follow-up (FU) studies are scarce, we aimed to provide insight into the evolutionary clinical and immunological scenario of unPAD children to adulthood and identification of biomarkers of primary immune deficiency (PID) persistence. Methods: A total of 23 pediatric unPAD patients underwent clinical and immunological FU for a mean time of 14 years (range 3–32 years, median 16 years). Results: UnPAD diagnosis may change over time. At the last FU, 10/23 (44%) children matched the diagnosis of transient hypogammaglobulinemia of infancy and 13/23 (56%) suffered from a persistent PID. In detail, an unPAD condition was confirmed in 7/23 (30%) patients, whereas 3/23 (13%), 2/23 (9%), and 1/23 (4%) were reclassified as common variable immunodeficiency, selective IgA deficiency, and isolated IgM deficiency, respectively. Low IgA, low specific antibody response to pneumococcus, and lower respiratory tract infections at diagnosis were independently associated with IEI persistence. Conclusions: Long-term monitoring of unPAD patients is required to define their outcome and possible evolution towards a definitive IEI diagnosis.

## 1. Introduction

Primary antibody deficiencies (PADs) consist of a varied group of conditions with different genetic etiologies characterized by an impairment of B cell development, differentiation or class switch recombination leading to hypogammaglobulinemia, and/or defective antibody production [1]. They represent the most common form of inborn errors of immunity (IEIs), counting for more than 60% of them [2]. PADs show a wide clinical spectrum, ranging from asymptomatic to severe forms. Common variable immunodeficiency disorder (CVID) represents the most frequent PAD, with a heterogeneous clinical phenotype, ranging from recurrent bacterial infections, mostly of the respiratory and gastrointestinal tracts to autoimmune disorders, allergy, lymphoproliferation, hyperinflammation, and/or malignancies [3]. Several other forms of milder PADs exist such as IgG deficiency, IgG subclass deficiency, selective IgA or IgM deficiency, and specific antibody defects. Some are in combination with each other [4,5,6,7]. In this context, a recent entity named “unclassified primary antibody deficiency” (unPAD) has been recognized and entered the European Society for Immunodeficiencies (ESID) definitions for clinical diagnosis. UnPAD patients match the following criteria: marked decrease in at least one of total IgG, IgG1, IgG2, IgG3, IgA, or IgM levels and/or failure of IgG antibody response(s) to vaccines, plus at least one of the following conditions: (I) recurrent or severe bacterial infections, (II) autoimmune phenomena (especially cytopenias), (III) polyclonal lymphoproliferation, (IV) affected family member. Secondary causes of hypogammaglobulinemia and clinical signs of T-cell-related diseases need to be excluded. UnPAD patients show a highly variable clinical spectrum as well [4,5,6]. Some children with unPAD may normalize their immunoglobulin levels within 4 years of age, framing the condition of transient hypogammaglobulinemia of infancy (THI); conversely, others may develop persistent or severe forms of PID. Although Ig levels might be less compromised in unPAD than in CVID, they may long remain unrecognized and undiagnosed. Moreover, they may suffer an underestimated risk of organ damage with a severe pulmonary involvement and a negative outcome. In fact, Janssen et al. reported that bronchial wall thickening, bronchiectasis, and atelectasis could be detected in 44%, 21%, and 19% of unPAD patients, respectively, similarly to CVID patients [7]. Immunoglobulin replacement therapy (IRT) is scarcely used in these patients, despite its efficacy being reported [1,8,9,10,11]. Recently, Karaman et al. found no significant difference in B-lymphocyte subset distribution of unPAD patients receiving Ig replacement therapy vs CVID patients [12]. In this study we provide a clinical, immunological, and genetic characterization of children with an early diagnosis of unPAD monitored for a mean time of 14 years (range 3–32 years, median 16 years) to outline their natural history and identify potential predictive and/or prognostic markers of final diagnosis.

## 2. Materials and Methods

Twenty-three pediatric patients (12–36 months) with an initial diagnosis of unPAD, attending the Pediatric Immunopathology and Allergology Unit/ Regional Referral Center for PIDs at Policlinico Tor Vergata in Rome, the Department of Pediatrics of University of Pisa, and the Pediatric Immunology Center of Federico II University in Naples were enrolled in the study. All patients matched ESID diagnostic criteria for unPAD (https://esid.org/Working-Parties/Registry-Working-Party/Diagnosis-criteria; accessed on 15 April 2023). A local ethical committee approved the study, and written informed consent was obtained from all participants or legal guardians.

According to general practice, immunological work-up of these patients included serum Ig levels by nephelometry, serum IgG subclass values by radial immunodiffusion, extended T and B cell immunophenotype by fluorescence-activated cell sorting (FACS), and specific IgG antibody response to tetanus and pneumococcal vaccines by ELISA (VaccZyme ^TM^ Tetanus toxoid IgG kit and VaccZyme ^TM^ Anti PCP- IgG, Binding Site, Birmingham, England). In patients with allergy, skin prick tests (SPT) and serum IgE (sIgE) were performed. Serum IgE was tested by ImmunoCAP FEIA, whereas airborne and/or food allergen extracts were used for SPT, according to clinical phenotype. In a limited number of patients, genetic analysis (next-generation sequencing—NGS) for the main PAD-associated genes was also performed. The NGS panel included the following genes: ICOS, TNFRSF13B, TNFRSF13C, TNFRSF12, CD19, CD81, CR2, CD20, CD27, IL21, IL21R, LRBA, CTLA4, PRKCD, PLCG2, NFKB2, NFKB1, PIK3CD, PIK3R1, PTEN, VAV1, RAC2, BLK, IKZF1, IRF2BP2, BTK, CD40L, SYK, LYK, FYK, MYD88, IRAK4, TNFSF13B, TNFSF17, TNFRSF17, RELB, REL, IKBE, IKBA, IKBB, IKK-alpha, IKK-beta, MAP3K14, RELA, STK4, AKT, LAT, IL12RB1, IL12B, IFNGR1, IFNGR2, ISG15, EVER1, EVER2, AICDA, CD40, UNG, CD79A, CD79B, PAX5, TCF3, BLNK. Sanger sequencing was used to confirm genetic variants detected by NGS as previously reported [13]. Clinical and immunological data were prospectively collected at enrollment and during follow-up (FU) at the following times: age 4 and then every 6–12 months according to common clinical practice. The last evaluation was performed on April 2023. The mean FU period was 14 years (range 3–32 years, median 16 years).

## 3. Statistical Analysis

Clinical and immunological data of patients were analyzed using Fisher’s exact test and the chi-squared test. A *p*-value < 0.05 was considered statistically significant. Univariate statistical analysis was performed using Graphpad Prism software version 8.2.1, whereas multivariate logistic regression data analysis was performed by Epi Info™ CDC software version 7.2 (available at https://www.cdc.gov/epiinfo/index.html; accessed on 15 April 2023).

## 4. Results

Twenty-three children with an initial diagnosis of unPAD, (14 males (61%) and 9 females (39%) were included in the study. Positive family history for PIDs was reported in 6/23 (26%) of patients, of whom two had parental consanguinity. A positive family history for early deaths was present in one patient (4%).

### 4.1. Clinical and Immunological Findings at Initial Diagnosis, According to Age (<24 or >24 Months)

At diagnosis, 22/23 (96%) unPAD patients (10/10 < 24 months and 12/13 > 24 months) were symptomatic. As detailed in Table 1, recurrent infections were the major clinical manifestations (22/22, 100%), mainly involving the upper and lower respiratory tract (URTI, LRTI) and the gastrointestinal and urinary tracts. Seven patients (32%) suffered from allergic conditions. Skin prick tests were positive in 6/7 (86%) patients, and three of them showed high sIgE levels. Only one patient, suffering from atopic dermatitis, had both normal sIgE and negative SPTs (data not shown). Autoimmune neutropenia was observed in 2/22 (9%) unPAD subjects. Clinical manifestations at diagnosis did not significantly differ in the two age groups. Isolated or combined IgG, IgA, and IgM defects (compared with age appropriate values) were detected in 82%, 61%, and 39% patients, respectively. Combined or isolated IgM defects were more frequent in older patients than in children <24 months (8/13, 62% > 24 months vs 1/10, 10% < 24 months, *p* = 0.028). Seven out of nine unPAD patients presented IgG subclass deficiency, with no correlation with age at diagnosis. A poor specific antibody response to tetanus and pneumococcus was detected in 4/18 (22%) and 7/19 (37%) patients, respectively. Standard immunophenotypic analysis was normal in all 23 patients. Extended B cell immunophenotyping was performed in 18/23 patients, and low expression of switched memory B cells and IgM memory B cells was observed in 10/18 (55%) and 1/18 (5%), respectively. Percentages of activated CD21 low B cells and transitional B cells were within age-matched reference values in all patients.

### 4.2. Clinical and Immunological Findings at 4 Years of Age and at Last FU (Mean 14 Years, Range 3–32 Years, Median 16 Years)

**Figure 1:**
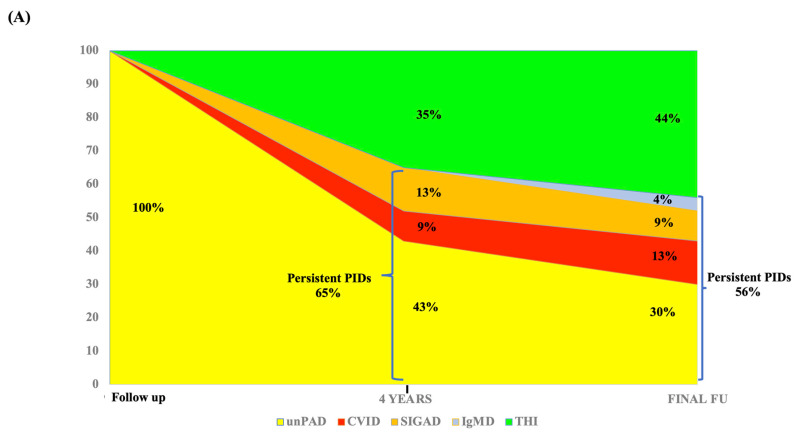

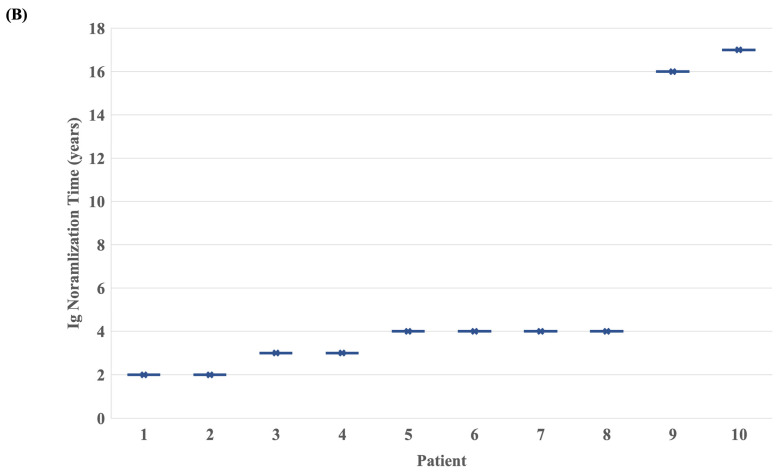
(**A**) Diagnostic reclassification of 23 unPAD patients at 4 years of age and at last FU (mean 14 years, median 16 years). Persistent PIDs: persistent primary immunodeficiencies; unPAD: unclassified primary antibody deficiency; CVID: common variable immunodeficiency disorder; SIGAD: selective IgA deficiency; IgMD: IgM deficiency; THI: transient hypogammaglobulinemia of infancy; Final FU: final follow-up. (**B**) Time of normalization of 10 THI patients.

All 23 patients underwent clinical and immunological follow-up every 6 months up to 4 years of age and then yearly for a mean time of 14 years (range 3–32 years, median 16 years). As reported in Figure 1A, at 4 years of age, immunoglobulin analysis revealed age-appropriated values in 8/23 (35%) patients, matching the diagnosis of THI. In the remaining 15 patients (65%), a persistent PID condition was observed. Overall, a diagnosis of unPAD was confirmed in 10/23 (43%), while 3/23 (13%) and 2/23 (9%) patients developed a selective IgA deficiency (SIGAD) and a CVID, respectively.

At the last follow-up, two patients reached age-appropriate Ig values at the age of 16 years and 17 years, respectively, joining the category of THI for a total of 10/23 (44%) patients (Figure 1A,B). Among the remaining 13 patients, 7/23 (30%) confirmed the clinical and immunological features of unPAD, whereas 3/23 (13%), 2/23 (9%), and 1/23 (4%) shifted to CVID, SIGAD, and isolated IgM deficiency, respectively. Clinical and immunological findings of patients with persistent PIDs compared to THI patients at the last FU (mean 14 years, median 16 years) are reported in Table 2. In line with Ig normalization, half of THI patients only suffered from allergic manifestations. Conversely, recurrent infections were only observed in the persistent PID group vs THI (5/13, 38% vs 0/10, 0%; *p* value 0.04), mostly with URTI and LRTI (3/5, 60% vs 0/10, 0%; *p* value 0.02). Moreover, isolated or combined IgG, IgA, and IgM defects were detected in 46%, 61%, and 54% of persistent PID patients, respectively, whereas associated IgG subclass deficiency was observed in 7/13 (53%) of them. A poor specific antibody response to tetanus and pneumococcus was found in 1/9 (11%) and 2/10 (20%) of persistent PID patients, respectively. When the extended B cell immunophenotype was performed in the persistent PID cohort, low switched memory B cells and low IgM memory B cells were detected in 7/13 (54%) and 2/13 (15%) of patients, respectively. The percentage of activated CD21 low B cells and transitional B cells were within age-matched reference values in all patients (data not shown).

### 4.3. Clinical and Immunological Findings at Diagnosis of Patients with a Definitive Diagnosis of Persistent PID vs. THI

To identify potential predictive and/or prognostic markers of clinical outcome, a retrospective analysis of clinical and immunological findings at diagnosis of 13 patients with a final diagnosis of persistent PID vs 10 patients with a final diagnosis of THI was performed. As reported in Table 3, LRTI were more frequently observed in patients with persistent PID than with THI (8/12, 67% PID patients vs 2/10, 20% THI patients, *p* value 0.03). Isolated or combined IgA deficiency and low anti-PCP antibody response were also found to be associated with a final diagnosis of persistent PID (11/13, 85% PID patients vs 3/10, 30% THI patients, *p* value 0.0013, and 7/11, 64% PID patients vs 0/8, 0% THI patients, *p* value 0.0128). The variables that showed a significant (*p* < 0.05) association with PID persistence in univariate analysis were evaluated in a logistic regression model for multivariate analysis. Low IgA, low specific antibody response to pneumococcus, and LRTI at diagnosis were observed to be independently associated with a persistent PID diagnosis.

### 4.4. Genetic Characterization

Next-generation sequencing analysis was performed in 8/13 (61%) of patients with persistent PID. Mutations in TNFRSF13B were identified in 4/8 patients (50%), belonging to two families. As reported in Figure S1, family A included three siblings, two sisters and one brother, in all of whom the final unPAD diagnosis was confirmed. A compound heterozygosis for two different TNF receptor superfamily member 13B (TNFRSF13B/TACI) gene mutations (TNFRSF13B c.301T>C plus TNFRSF13B c.204dupA) was identified in the two sisters and a heterozygous TNFRSF13B c.301T>C mutation in the brother. Their mother carried a TNFRSF13B c.301T>C mutation with the absence of clinical and immunological abnormalities; their father carried a TNFRSF13B c.204dupA and presented an asymptomatic isolated IgM defect. Regarding the clinical picture, both sisters suffered from both URTI and LRTI, whereas the brother had a milder phenotype. A heterozygous TNFRSF13B c.301T>C mutation was also detected in another patient with a final diagnosis of CVID. This patient showed a positive family history for early deaths of unknown-causes and a personal clinical history of URTI, LRTI, and gastrointestinal infections since the age of 9 months. Due to infectious recurrences, he initially received antibiotic prophylaxis and later immunoglobulin replacement therapy. His father carried the same mutation and was suffering from Hashimoto thyroiditis, psoriasis, and recurrent oral aphthosis.

## 5. Discussion

The onset and clinical spectrum of unPAD patients is widely heterogeneous, and knowledge of their natural history is still scarcely investigated. Some children might be identified at an early age and initially diagnosed as unPAD to enter the THI definition when normalization of their immunoglobulin levels occurs within 4 years of age. Some others may develop over time a defined antibody defect, reaching the definitive diagnosis of a classified IEI. However, at unPAD diagnosis, no markers are currently identified to allow the distinction between patients who will achieve a condition of transient hypogammaglobulinemia from those who will persist in the same condition or will develop other humoral defects, which would be crucial to timely initiate appropriate monitoring and treatment.

The aim of our study was to analyze the clinical, immunological, and genetic characterization of a cohort of children who received an early diagnosis of unPAD and their long-term clinical and immunological monitoring and outcome. To our knowledge, this is the first prospective study describing the evolution of unPAD children towards adulthood, with the clinical and immunological evaluation of their long-term follow-up.

Except for one asymptomatic child, all symptomatic children suffered from recurrent infections, mostly of the respiratory tract, and to a lesser extent from allergy and autoimmunity (32 and 9%, respectively). Indeed, as shown for other PAD patients [6,14,15,16,17,18,19,20,21], in unPAD children, recurrent respiratory infections represent the clinical hallmark, particularly in the first decade of life [18]. Conversely, immune dysregulation may complicate the clinical course mostly in early and late adulthood [14,18]. In the overall cohort, we observed that at diagnosis, an isolated or combined IgG defect was more frequently observed than an isolated or combined IgA defect. Instead, an isolated or combined IgM defect was more frequently observed in children >24 months of age than at a younger age.

In a previous paper, we reported that in children with an initial suspicion of THI, nowadays pertaining into the category of unPAD, a milder clinical and immunological profile and a benign course over time was observed in comparison to those children who did not normalize their IgG levels [22]. In the present study, at 4 years of age, 8/23 (35%) patients matched the diagnosis of THI, and, at last follow-up, two more patients (44% of the total cohort) enriched the THI category at 16 and 17 years of age, respectively. In accordance with previous studies by us and other groups [22,23], IgG normalization occurs more frequently, despite not doing so exclusively, within the first 4 years of age. Indeed, there is no univocal evidence in literature on the time to recovery and Ig normalization that might occur until the third or fourth decade of life [23]. In line with IgG normalization, THI patients maintained a milder clinical picture, mostly allergic manifestations, whereas patients with persistent quantitative and/or qualitative antibody defects featured recurrent/severe infections, mostly of the lower respiratory tract. At final follow-up, 13 patients (56%) suffered from a persistent PID. In detail, an unPAD condition remained in 7/23 (30%) patients, whereas some moved to different primary antibody defects, comprising CVID (3/23; 13%), SIGAD (2/23; 9%), and isolated IgM deficiency (1/23; 4%). Interestingly, one patient who, at enrollment, matched the clinical diagnostic criteria for unPAD, moved to a SIGAD condition at 4 years of age to later develop a CVID at 5 years of age. This patient, harboring a C104R (TNFRSF13B c.301T>C) heterozygous TACI variant, who continued to suffer from recurrent and severe infections, firstly required antibiotic prophylaxis and, after receiving a CVID diagnosis, benefited of additional immunoglobulin replacement therapy. The multi-step diagnostic evolution of this patient clearly outlines the wide spectrum of antibody disorders associated with TACI mutations, as previously reported [24]. Mutations in TNFRSF13B have been identified in a family comprising three siblings, two sisters and one brother, who maintained the unPAD diagnosis. Particularly, the two sisters carried a compound heterozygosis for two different TNFRSF13B gene mutations (TNFRSF13B c.301T>C plus TNFRSF13B c.204dupA), whereas a heterozygous TNFRSF13B c.301T>C mutation was found in the brother. Their mother carried the TNFRSF13B c.301T>C mutation, and their father the TNFRSF13B c.204dupA. The same compound heterozygosis found in the two sisters has been previously reported by Salzer et al. [25] and associated with normal expression of switched and IgM memory B cells, as in our female patients.

Moreover, the asymptomatic unPAD patient who was diagnosed at 15 months, whose brother was suffering from CVID, over time developed recurrent respiratory infections, receiving the final diagnosis of a selective IgM deficiency. It is still unclear why some patients may be asymptomatic. Functional immunological factors as well as epigenetic or environmental factors might be compensative for a certain time.

When we investigated at diagnosis potential markers of dissection between PID persistence vs THI, we observed that in addition to LRTI, low IgA and low specific antibody response were independently associated with a persistent condition. Conclusively, we demonstrated that a subgroup of children with unPAD shares common B cell abnormalities with patients suffering from a range of antibody defects, mostly CVID, according to previous reports [12,26,27]. We point out that a critical long-time clinical and immunological follow-up of unPAD patients is recommended to monitor their evolution (towards a definitive antibody defect in most cases), which may be variable over time, and also among family members with the same genetic mutation, as herein described. As the reservoir for CVID lacks molecular definition, the identification of unPAD patients potentially evolving to a CVID diagnosis during the routine monitoring process is of relevance. UnPad patients are usually underestimated and often suffer from a long diagnostic lag and delayed optimal management. However, a significant proportion of unPad patients may emerge with pulmonary and extrapulmonary complications that might affect several aspects of life. Similarly to CVID patients, it has been reported that bronchial wall thickening, bronchiectasis, and atelectasis could be detected in 44%, 21%, and 19% of unPAD patients, respectively [7]. Their autoimmune and inflammatory complications and whether they might be addressed with immunoglobulin replacement therapy are largely unknown, although it has recently been reported that B-lymphocyte subset distribution did not significantly differ between unPAD patients who received Ig replacement therapy from those with CVID [8]. The lack of extensive and comparable studies describing unPAD cohorts hampers the draft of international networks for the assessment of their diagnostic approach and monitoring. Although the progressive advances in genetic and functional technology will allow for the identification of IEI disorders currently assembled in the unPAD reservoir, increasing awareness for their early diagnosis, the molecular pathways and epigenetic changes underlying the complex heterogeneity of unPAD disorders deserve further investigation.

## 6. Conclusions

Our study, despite the limited cohort, is the longest FU study analyzing a pediatric cohort of unPAD patients. A continuous long-term clinical and immunological monitoring of unPAD children is necessary to define their outcome and possible evolution towards a definitive IEI defect. We focused on distinct clinical-immunological markers, suitable for identifying patients at higher risk of PID persistence. Furthermore, as the genetic and functional characterization of patients with persistent hypogammaglobulinemia might provide us with valuable information on the pathogenic role of distinct molecules, potentially indicating specific treatment, we emphasized the need to structure national and international networks for the diagnostic approach and monitoring of the heterogeneous condition of unPAD patients.

## Figures and Tables

**Table 1:** Clinical and immunological findings of 23 patients with an initial diagnosis of unPAD, according to age at diagnosis (<24 and >24 months).

	unPAD	<24 Months (10 pts)	>24 Months (13 pts)	*p*-Value
CLINICAL MANIFESTATIONS	22/23 (96%)	10/10 (100%)	12/13 (92%)	ns
Infections	22/22(100%)	9/10 (90%)	12/12 (100%)	ns
URTI	12/22 (55%)	6/10 (60%)	6/12 (50%)	ns
LRTI	10/22 (45%)	4/10 (40%)	6/12 (50%)	ns
GI	5/22 (23%)	1/10 (10%)	4/12 (33%)	ns
UTI	5/22(23%)	4/10 (40%)	1/12 (8%)	ns
FEVER	1/22 (5%)	1/10 (10%)	0/12 (0%)	ns
SKIN	1/22 (5%)	1/10 (10%)	0/12 (0%)	ns
Allergy	7/22 (32%)	3/10 (30%)	4/12 (33%)	ns
Asthma	2/7 (29%)	0/3 (0%)	2/4 (50%)	ns
Atopic dermatitis	3/7 (43%)	2/3 (67%)	1/4 (25%)	ns
Food allergy	2/7 (29%)	1/3 (33%)	1/4 (25%)	ns
Conjiunctivitis	1/7 (14%)	1/3 (33%)	0/4 (0%)	ns
Rhinitis	4/7 (57%)	1/3 (33%)	3/4 (75%)	ns
Autoimmunity	2/22 (9%)	2/10 (20%)	0/12 (0%)	ns
Neutropenia	2/2 (100%)	2/2 (100%)	-	ns
IMMUNOLOGICAL ABNORMALITIES	23/23 (100%)	10/10 (100%)	13/13 (100%)	ns
Isolated or combined IgG defect	19/23 (82%)	9/10 (90%)	10/13 (77%)	ns
Isolated or combined IgA defect	14/23 (61%)	4/10 (40%)	8/13 (62%)	ns
Isolated or combined IgM defect	9/23 (39%)	1/10 (10%)	8/13 (62%)	0.028
Combined IgG defect	13/23 (56%)	6/10 (60%)	7/13 (54%)	ns
Combined IgA defect	13/23 (56%)	5/10 (50%)	8/13 (62%)	ns
Combined IgM defect	8/23 (35%)	0/10 (0%)	8/13 (62%)	0.0075
IgG subclasses defect	7/9 (77%)	1/2 (50%)	6/7 (86%)	ns
Low anti TT antibody response	4/18 (22%)	3/7 (43%)	1/11 (9%)	ns
Low anti PCP antibody response	7/19 (37%)	2/7 (29%)	5/12 (16%)	ns
Low switched memory B cells	10/18 (55%)	5/7 (71%)	5/11 (45%)	ns
Low IgM memory B cells	1/18 (5%)	1/7 (14%)	0/11 (0%)	ns

URTI upper respiratory tract infections; LRTI lower respiratory tract infections; GI gastrointestinal infections; UTI urinary tract infections; TT tetanus toxoid; PCP pneumococcal.

**Table 2:** Clinical and immunological findings of 13 persistent PIDs patients vs 10 THI patients at last FU (mean 14 years, median 16 years).

	Persistent PIDs (13 pts)	THI (10 pts)	*p*-Value
CLINICAL MANIFESTATIONS			
Infections	5/13 (38%)	0/10 (0%)	0.04
URTI	3/5 (60%)	-	0.02
LRTI	3/5 (60%)	-	0.02
GI	1/5 (20%)	-	ns
Allergy	6/13 (46%)	5/10 (50%)	ns
Asthma	2/6 (33%)	0/5 (0%)	ns
Rhinitis	3/6 (50%)	4/5 (80%)	ns
Conjiunctivitis	1/6 (17%)	2/5 (40%)	ns
Autoimmunity	2/13 (15%)	0/10 (0%)	ns
Neutropenia	2/2 (100%)	-	ns
Vasculitis	1/2 (50%)	-	ns
IMMUNOLOGICAL ABNORMALITIES	13/13 (100%)	0/10 (0%)	0.0001
Isolated or combined IgG defect	6/13 (46%)	0/10 (0%)	0.02
Isolated or combined IgA defect	8/13 (61%)	0/10 (0%)	0.003
Isolated or combined IgM defect	7/13 (54%)	0/10 (0%)	0.007
IgG subclass defect	7/13 (54%)	0/10 (0%)	0.007
Low anti TT antibody response	1/9 (11%)	0/10 (0%)	ns
Low anti PCP antibody response	2/10 (20%)	0/10 (0%)	ns
Low switched memory B cells	7/13 (54%)	0/10 (0%)	ns
Low IgM memory B cells	2/13 (15%)	0/10 (0%)	ns
Genetic Characterization	8/13 (61%)	-	
TNFRSF13B mutations	4/8 (50%)		
TNFRSF13B c.301T>C plus TNFRSF13B c.204dupA	2/4 (50%)		
Heterozygous TNFRSF13B c.301T>C mutation	2/4 (50%)		

URTI upper respiratory tract infections; LRTI lower respiratory tract infections; GI gastrointestinal infections; UTI urinary tract infections; TT tetanus toxoid; PCP pneumococcal.

**Table 3:** Clinical and immunological findings at diagnosis of patients with a final diagnosis of persistent PID vs. THI.

	Persistent PIDs (13 pts)	THI (10 pts)	Univariate Analysis *p*-Value	Logistic Regression *p*-Value
Positive Family History for PID	5/13 (38%)	1/10 (10%)	ns	
CLINICAL MANIFESTATIONS				
Infections	12/13 (92%)	10/10 (100%)	ns	
LRTI	8/12 (67%)	2/10 (20%)	0.04	<0.05
Allergy	4/13 (31%)	3/10 (30%)	ns	
Autoimmunity	1/13 (8%)	1/10 (10%)	ns	
IMMUNOLOGICAL ABNORMALITIES				
Isolated or combined IgG defect	11/13 (85%)	8/10 (80%)	ns	
Isolated or combined IgA defect	11/13 (85%)	3/10 (30%)	0.0013	<0.05
Isolated or combined IgM defect	6/13 (46%)	3/10 (30%)	ns	
IgG subclass defect	5/5 (100%)	2/4 (50%)	ns	
Low anti PCP antibody response	7/11 (64%)	0/8 (0%)	0.0128	<0.05
Low switched memory B cells	7/13 (54%)	0/5 (0%)	ns	
Low IgM memory B cells	1/13 (8%)	0/5(0%)	ns	

LRTI lower respiratory tract infections; PCP pneumococcal.

## Data Availability

The data are available upon request from the corresponding author.

## Supplementary Materials

The following supporting information can be downloaded at: https://www.mdpi.com/article/10.3390/jcm12134206/s1, Figure S1: Pedigree analysis of patients with TACI mutations.

## Abbreviations

CVID	common variable immunodeficiency
ELISA	enzyme-linked immunosorbent assay
ESID	European Society for Immunodeficiencies
FACS	fluorescence-activated cell sorting
FU	follow-up
GI	gastrointestinal infections
IEI	inborn error of immunity
IgMD	IgM deficiency
IRT	immunoglobulin replacement therapy
LRTI	lower respiratory tract infection
NGS	next-generation sequencing
PAD	primary antibody deficiency
PID	primary immunodeficiency
PCP	pneumococcal
SIGAD	selective IgA deficiency
sIgE	serum IgE
SPT	skin prick tests
THI	transient hypogammaglobulinemia of infancy
TNFRSF13B/TACI	TNF receptor superfamily member 13B
TT	tetanus toxoid
UnPAD	unclassified primary antibody deficiency
UTI	urinary tract infections
URTI	upper respiratory tract infections
