# Supplementary figures and images for: The Evolutionary Scenario of Pediatric Unclassified Primary Antibody Deficiency to Adulthood
Source: J Clin Med. 2023 Jun 22;12(13):4206. doi: 10.3390/jcm12134206 (PMC10342284; doi:10.3390/jcm12134206)

Supplementary Figure S1

Family A

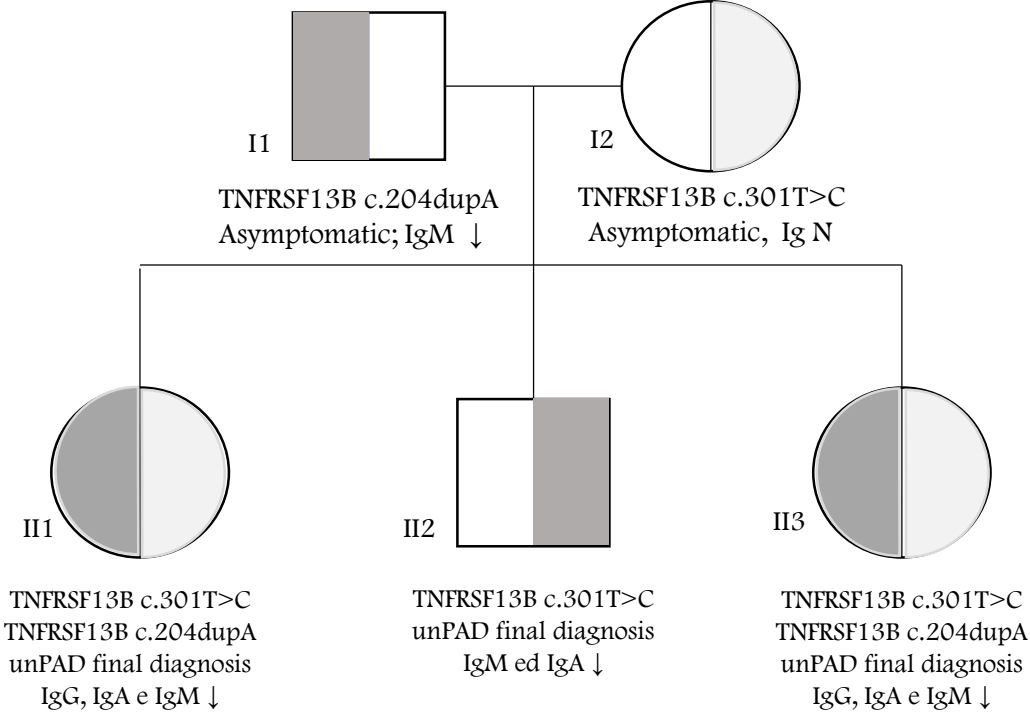

Family B

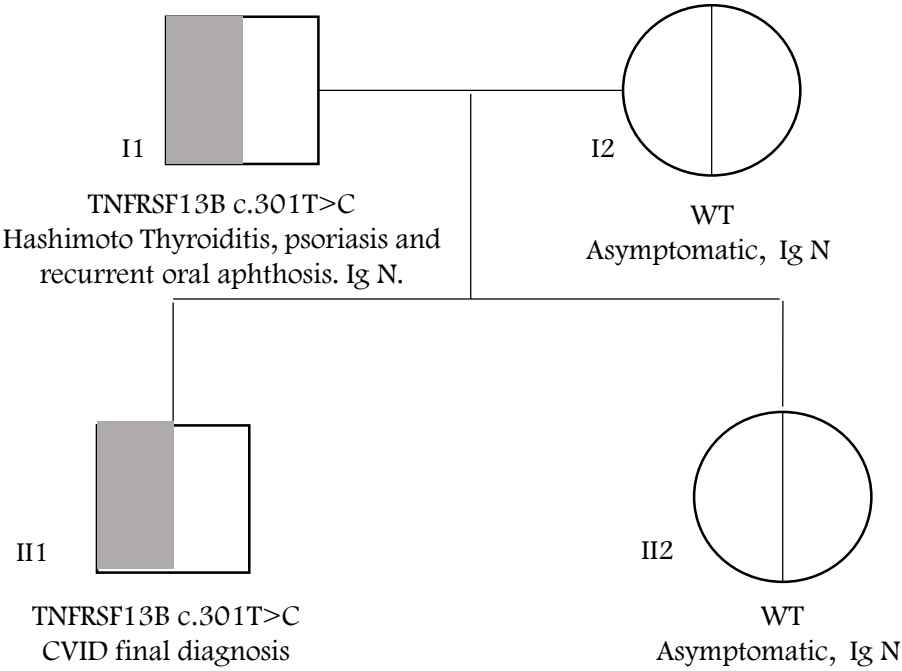

Supplement: Supplementary file 1 [file jcm-12-04206-s001.zip › jcm-2430208-supplementary.pdf]
